# Food Systems Development: The Necessary Paradigm Shift for a Healthy and Sustainable Agrarian Transition, a Case Study from Bougainville, Papua New Guinea

**DOI:** 10.3390/ijerph19084651

**Published:** 2022-04-12

**Authors:** Genia Hill, Rachel Friedman, Paul Dargusch

**Affiliations:** 1 School of Earth and Environmental Sciences, University of Queensland, Brisbane, QLD 4072, Australia; p.dargusch@uq.edu.au; 2 Institute for Climate, Energy, and Disaster Solutions, Australian National University, Canberra, ACT 0200, Australia; rachel.friedman@anu.edu.au

**Keywords:** global change, food systems, agricultural development, Melanesia, gender, Papua New Guinea

## Abstract

Classical agricultural development paradigms prioritise basic requirements such as agronomic, caloric and economic needs for the target environment and for beneficiaries. As challenges associated with climate change, globalisation, and population growth compound and amplify one another, project scope must be broadened to take a holistic food systems approach that includes sociocultural and historical contexts, as well as climate impacts as underpinning project design. In this paper, we illustrate the importance of adopting a food systems development paradigm rather than a classical agricultural development paradigm through a case study in Bougainville, Papua New Guinea. The case uses Rich Picturing, targeted and focus-group interviews, and garden visits in remote Bougainville; it provides a poignant illustration of the importance of this more holistic perspective given the historical inefficacy of food systems development, as well as Papua New Guinea’s exposure to a plethora of compounding environmental, social, economic, and political stresses and shocks that demonstrate the important linkages between ecosystem services and health. The study aims to demonstrate how including localised gender dynamics, climate vulnerability, rapidly morphing social norms, and climate analogue environments is critical in building food systems resilience and is key to designing policies, programs, and development projects that more effectively address environmental, sociocultural, and health considerations. Building on the inadequacies in agricultural development efforts previously documented for Papua New Guinea, we propose an improved framing for food systems development and identify areas for future research.

## 1. Introduction

Food connects human health, environment, and livelihoods. Its production is a key area for improvement in sustainable development. Globally, food supply chains are responsible for 31% of greenhouse gas emissions [1]. Yet, they also provide income for a third of the world’s population (largely in the Global South) and are a fundamental determinant of both human and environmental health. In Melanesia, smallholder farming systems have historically provided livelihoods and sustenance for the vast majority of the population through low input and sustainable farming practices [2]. Over time, however, this model of food production has faced growing pressures associated with agrarian transition and global environmental change. Papua New Guinea (PNG), Melanesia’s largest country, faces a plethora of shocks and stresses in its food system, and it ranks among the most vulnerable to climate change in the world when adaptive capacity is considered [3]. Hence it is vital that food systems in the region are developed that ensure a healthy population and environment. In this study, we focus on Bougainville, PNG, a case study within the Melanesian context. The case of Bougainville illustrates the need for an improved approach to food production and consumption, particularly given the historical insufficiency of food systems development (including policy and aid projects) in the region.

Food systems encompass a broad range of activities and sectors, yet this is not always reflected in how resources are directed. “Food systems” conceptualisations consider the interactions between and within bio-geophysical and human environments, activities along the food supply chain from production to consumption, and outcomes of those activities (such as food security and social welfare) [4]. Further, food systems are shown to link environment and health, providing “*healthy, adequate, affordable and safe diets, which are the basis of a healthy life and the pre-condition for successful participation in society of each individual, while safeguarding the clean and healthy planet…*” [5]. While food systems are often expected to achieve diverse objectives relating to health, livelihoods, and even biodiversity, development projects have often taken the form of ‘classical agricultural development’ and cash cropping. We define ‘classical agricultural development’ as “the process that creates the conditions for an increase in the level and rate of agricultural productivity, such that agricultural potential is fulfilled“ (modified from Krishna, 1992 and OECD, 2006) [6,7].

Food systems development in PNG has had a limited scope of focus, and aid interventions and policy changes have been insufficient, with GDP per capita stagnating over the last 40 years and income inequality continuing to grow [8]. An assessment of foreign aid and its contribution to economic growth in PNG between 1965 and 1999 found little evidence to support a positive relationship between aid and economic growth [9]. Further, Hughes (2010) states that the region is a complete development failure, arguing that there are irrelevant, inappropriate, or corruptly structured development targets that are supported by irrelevant Millennium Development Goals [10]. These are inappropriate in the Pacific context, as traditional subsistence gardens are largely more productive than post-intervention environments [10].

The pressures on food production systems are highly complex. Global environmental change (GEC), which encompasses climate change, globalization, and population growth, presents in the Melanesian context as changes to the hydrological cycle, increasing mean temperature, and soil salination (due to climate change), land pressure, bush meat or fish stock decline, soil degradation due to reduced swidden times, and changes in land tenure due to land ownership formalisation (driven by population growth and globalization impacts) [2,11,12]. These can be characterised as “shocks” or “stresses”. Shocks are those events that occur over the short-term and stresses are sustained events that occur incrementally over time. Examples of these in Melanesian food systems are shown in Table 1. Pacific food systems have become increasingly vulnerable to shocks and stresses, which impact the production, distribution, and acquisition of food; this is extenuated by the impacts of climate change as well as the impacts of COVID-19 [13].

**Figure 1:**
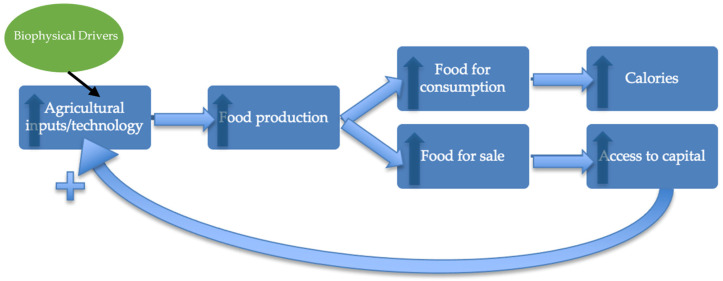
The classical agricultural development paradigm focuses on increasing food production for the purpose of generating calories and income [26,27]. This process is relatively linear, neglecting those feedbacks or responses relating to culture or context. The arrows demonstrate feedbacks within this linear model, whereby an increase in inputs results in an increase in food production, and eventually in an increase in calories and capital, which can further be invested in increased inputs/technology.

**Figure 2:**
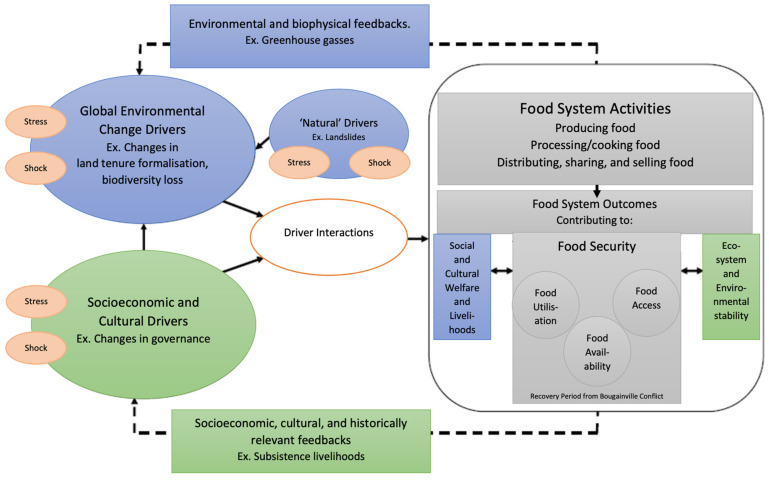
Figure adapted from Ericksen (2008) and applied to a Bougainvillean context. It represents the interconnections and feedbacks of the food system as a whole, addressing all components of the food system (and what is considered “within its boundaries”) along the supply chain (food system activities) and with reference to sociocultural and global environmental change drivers, shocks, and stresses, not only the biophysical as with the prevailing classical development paradigms [4].

The classical agricultural development paradigm has resulted in minimal gains for PNG in recent years, and focuses on basic requirements such as agronomic, caloric, and economic needs for the target environment and for beneficiaries. Although projects have become theoretically more inclusive (closer to a “systems approach”) in recent years, this change largely has not been operationalised in the Melanesian and Pacific context [16]. Here we posit the need to shift from an “agricultural development” framework (see Figure 1) to an inclusive and contextualised “food systems” framework (see Figure 2) and in turn, a “food systems development paradigm” for improved development.

We propose that using an established food systems approach in policy and development project design will better reflect the growing complexity of challenges facing food production systems, historical aid inefficacy and the need for resource efficiency in development. The concept of ‘food systems’ and its definition have been rising in prominence in recent scholarship, as has its application to development. The consideration of influences outside of bio-physical or GEC drivers is also rising in prominence [4]. Food systems frameworks that include metrics for ‘climate and environment’, ‘nutrition and health’, ‘food security’, ‘social welfare’, and ‘food economy’ are promoted by the Sustainable Development Goals (2015), Bènè et al. (2019), Fanzo et al. (2020), Hebinck et al. (2021), Mayton et al. (2020), and Melesse et al. (2020) [5,17,18,19,20,21]. Hebinck et al. (2021) demonstrate that earlier works tend to exclude principles of food security or food economy, such as Chaudhary et al. (2018), Jones et al. (2016), and Le Vallée et al. (2016) and over time, frameworks are becoming more inclusive in their approach [5,22,23,24]. The importance of historical and cultural context receives less attention. Academic literature has used ‘appropriate localization’ as a proxy for history and culture, such as in Bènè et al. (2019), Caron et al. (2018), and Hebinck et al. (2021) [5,18,25]. These factors have rarely been expressly called out, as they are in the current paper.

We use a case study of subsistence-based food systems in Bougainville, PNG to illustrate the need for the proposed more-holistic development paradigm. We propose the more-inclusive framing will better facilitate healthy and sustainable development outcomes by including factors such as sociocultural or historical appropriateness in development project design.

## 2. Materials and Methods

### 2.1. Conceptual Framing

Figure 2 draws on Ericksen (2008) and additional models to present a holistic and contextualized approach to analysing food systems, including elements of the frames posited by Addinsall et al. (2015) and Jackson et al. (2017) [4,28,29]. Addinsall et al. (2015) situate agricultural development within a culturally appropriate framework, acknowledging that in the Pacific context, subsistence agriculture is not only a livelihood, but is also culturally significant, exhibiting elements of natural, human, financial, social, and physical capitals [26]. For instance, food systems in which labour is shared among clans or families have elements of both human and social capital. Those in Melanesia experience a unique combination of stressors, including climate change, urbanisation, and modernisation of land tenure that was previously informally titled [30]. This framing acknowledges the importance of working across disciplines such as sociology, economics, agronomy, and ecology to address these challenges.

In their *Framework for Disaster Vulnerability*, Jackson et al. (2017) build on existing post-disaster vulnerability frameworks by accounting for historical context, and apply this to a case study in Ni-Vanuatu [29]. The framework emphasises how historical context interplays with disaster context (in their influence on one another) [29]. This is particularly relevant to the Bougainvillean post-conflict social context. In addition to addressing socio-economic or biophysical vulnerabilities as in the traditional disaster susceptibility frameworks, Jackson et al. posit that population growth, resource diminishment, resource-dependent livelihoods, and changing lifestyles should also be considered as underlying vulnerability [29].

We use these two models to understand points of resilience and vulnerability in the case study food system by viewing the system through lenses of sociocultural and contextual significance and disaster vulnerability. In using this interdisciplinary and contextualised approach, we employ the “Food Systems Development Paradigm” and identify strategies to increase resilience and improve healthy and sustainability across the food system.

### 2.2. Data Collection

We applied various methods to collect and triangulate food system data from the field, including rich picturing, garden visits, and focus group interviews. Three wards were visited, though they have been anonymised due to the vulnerable nature of these peoples and in accordance with institutional ethics approval.

### 2.3. Study Site

The study site is located in the Autonomous Region of Bougainville (“Bougainville”), a group of islands in the east of PNG. Bougainville’s recent history has been characterised by violence and a long struggle for sovereignty that culminated in the Bougainville Conflict (1988–1998). While there were many factors contributing to the Bougainville Conflict, the environmental impacts from the Panguna Mine are often considered its catalyst [31]. It is estimated that upwards of 10% of the population of Bougainville died in the Bougainville Conflict due to violence, starvation, or lack of medical resources—a large part of which can be attributed to a blockade that impacted most of the region through the 1990s [32,33]. Local economic activity was effectively halted due to the conflict, and the region has not recovered despite two decades of rebuilding [33].

In late 2019, Bougainvilleans voted by 98.31% for independence from PNG. Moving toward independence requires establishing “fiscal self-reliance” and “good governance” as described in the Bougainville Peace Agreement [34]. There are two popular approaches to Bougainville’s future development: mining, or alternatives such as agriculture and sustainable tourism, which are less profitable in the short term [32,33,35]. This places Bougainville at the beginning of a transition period that will impact politics, infrastructure, and development pathways, and may result in increased socio-political tensions in the region. It also presents an opportunity to develop sustainable and resilient practices and policies that contribute to Bougainville’s long term financial independence, sovereignty and ecosystem health.

The case discussed in this paper is a remote region on the West Coast of Bougainville, roughly 100 km from the Panguna Mine.

It has limited infrastructure: no roads, postal services, waste management, electricity, or running water. Within the community there is an elementary and primary school, a small community health centre, dirt walking paths, and a police service. The study area catchment is roughly 200 km^2^ and is covered in rainforest or anthropogenically altered land for food gardens, cocoa or coconut plantations, walkways, and hamlets. In the Melanesian context, ‘food gardens’ are small plots of land operated on a familial basis and used largely for subsistence crop production. The wet season runs November to May and the dry season runs June to October, around which agricultural activities are planned. Climate modelling projects that Bougainville and much of Melanesia will become both warmer and wetter with climate change [36,37].

### 2.4. Field Methods

The lead author used rich picturing to pique community interest in the research project and initiate discussion and data collection in the case study area. Rich picturing was developed as a problem sketching tool [38] and is particularly useful in complex situations where it is important to minimise facilitator bias [39,40]. Roughly 50 participants formed groups of 8 to 10 individuals and were prompted to sketch their food system, including food acquisition, cash cropping, and process flows, as well as any challenges they face in this system. The researchers intentionally left the prompt vague to facilitate creativity and eliminate bias among respondents. These data were collected by the researchers and analysed in NVIVO to identify common themes.

Data were also collected in the form of stories conducted with groups of between 3 and 15 members composed primarily of adult women. Bougainvilleans define conversations as “telling stories”, which fits well within the theoretical framing of storytelling methodology [41]. Women were the target group, but as community interest in the project grew, more men became involved. Participants were informed of the researcher’s presence in the community prior to arrival. The researcher and field assistants walked to various community areas in the region and convened groups in a central hamlet in each area. In this study, participants would typically be segregated by age into two or three groups and directed to complete the consent process and then to tell stories. Researchers asked questions regarding the composition of the food system, gender roles in agricultural decision making and food consumption, changes to the food system during the Bougainville Conflict and over time, and challenges to food security and the associated existing resilience strategies. While questions were systematically scripted, the “go along” approach was employed in vivo (see Appendix A for guiding questions). This allowed participants to drive the conversation and to tell stories in an ad hoc manner, and for the researcher to ask follow-up questions.

Each session was followed by an open discussion and question and answer session (Q & A) where global environmental change (particularly climate change and population pressure) and potential future resilience strategies were discussed. Each series of storytelling focus groups and Q & As were then followed on with several hours of cooking using both traditional and quotidian methods, culminating in a group prayer and meal. There were nine focus group interviews and numerous informal conversations with local health workers, teachers, elected community leaders, and government appointed officials. Most groups had at least one strong English speaker, though occasionally a local field assistant translated where needed. Conversations were recorded, transcribed, and coded. Additionally, a detailed journal of daily observations was recorded by the primary researcher.

Finally, a series of gardens in each ward were visited and the garden owner described their gardening practices. We asked scripted questions about agricultural practices and decision-making, though the ethnographic “go along” method was also used during this discussion. Between two and four gardens were visited in each ward, and their composition was roughly drawn or photographed and recorded with a digital recorder.

Data were transcribed and then coded using NVIVO qualitative data analysis software to determine themes and nodes [41,42]. The approach of inductive and deductive thematic analysis to discern themes was adopted from Fereday and Muir-Cochrane (2006) [42]. Theoretical (deductive) codewords gleaned from the literature, such as “basic composition”, “challenges”, and “conflict” as well as emerging (inductive) codes were derived from common words that arose in respondent answers, such as “continuous rain” and “transportation”.

The University of Queensland Institutional Human Research Ethics Committee A approved these methods under application number 2018001909.

## 3. Results

In the exploratory portion of this study, it became clear that some development efforts had neglected context. These oversights are illustrated in the results of this paper, which are presented according to the food systems model depicted in Figure 2. Aspects of the food system are presented as activities and outcomes of the system. The important biophysical and environmental elements are discussed, as are the socioeconomic, cultural and historical components of the food system (including the role of gender in food system activities and outcomes and recovery from the Bougainville conflict). Stresses and shocks on the system, as well as the system’s vulnerability and existing resilience strategies, are also discussed.

### 3.1. Food System Activities and Outcomes

The food system in the case study area is primarily subsistence-based, fished and foraged with minor supplementation of “shop food”, a small amount of cash cropping, and few commercial components (see Supplementary Table S1). Staple crops are sweet potato (*kaukau*), taro, cassava, banana, coconut, and yam, with other common foods including peanuts, snake beans, tomato, capsicum, sugarcane, and pit-pit (*Saccharum edule*, an edible grass). Collection of wild plants, primarily Posu’e, a foraged fern leaf, is common; occasionally it is eaten with prawns or chicken. Wild plants are additionally used for medicinal and practical purposes (such as food storage). Food is often shared within clan groups, particularly during the hunger season or with those who do not have their own substantial gardens (such as the community schoolteacher). Despite a wide variety of food sources, participants in this study all cited food shortages in the last year.

Gardens are variable in size but always composed predominantly of sweet potato (5–10 varieties) planted in blocks of varying maturity. Cassava (>5 varieties) and bananas (5–10 varieties) surround the perimeter of most gardens. Some gardens also have blocks of peanut (3–5 varieties) and irregular plantings of vegetables. Pit-pit (an edible grass) and coconut occasionally surround the gardens with the banana and cassava blocks. Peanuts and beans are used in some gardens to improve soil quality and are often sold in the market. One trial garden interplants marigolds with taro to improve pest resistance, though in most gardens pests are not usually removed manually or using pesticides. Pests are not problematic in new gardens but become an issue after one to two years. About half of households keep chickens near the home (though away from food preparation areas).

Crops are predominantly propagated from cuttings, rather than using commercial inputs such as seeds, fertiliser, or pesticides (often due to cost). Cuttings needed for new gardens are sourced from the previous garden or from a family member or close friend.

Excess garden crops are shared or sold in the market, while cash crops (primarily cacao, copra, and betel nut) are sold in the capital. Monetary income is used to purchase shop food, school fees, and healthcare. Foods most often purchased from shops (either a local unregistered “trade store” or in the capital), include rice, salt, sugar, coffee, and tinned fish. Other common shop purchases are soap and clothing. Food sharing is frequent and casual, with many respondents reflecting the sentiment in focus group discussions: “*If we have extra food, when someone is short of food, we share food with them*”.

### 3.2. Drivers Influencing the Food System

#### 3.2.1. Socioeconomic, Cultural and Historically Relevant Components of the Food System

The main socioeconomic challenges cited in the food system are lack of transport access to sell garden food and cash crops and the prohibitive price of agricultural inputs for cash crops. The most frequently cited changes in the case study food system over time were the rise of shop food consumption (gradually over the lifetimes of the oldest participants, who were in their 70s, and with a pause during the Bougainville Conflict), increase in garden size due to larger families, increase in reliance on sweet potato, and reduced access to markets in the capital city and to cash crop buyers due to a bridge failure in the 1980s.

The introduction of metal tools for gardening occurred in the late 1960s to early 1970s. Prior to these, large sticks with sharp carved logs known as “*karioki*” were used to plant and harvest sweet potatoes and taro. Application of chemical inputs had not increased due to their prohibitive price.

A respondent over the age of 60 characterised the most widespread change in daily diets as being the increased reliance on sweet potato: “*We grow more kaukau (sweet potato) now, since it was introduced. It is ready faster than taro and is easier to grow. Now [we eat] more kaukau. At a small age we ate only taro and yam. We also had more protein, like pigs, in the past, now we nogat (don’t)*”.

Historically relevant factors in the food system include long-lasting impacts from the Bougainville Conflict (explored in the following section), as well as the persistent impacts of aid projects. For instance, a large NGO facilitated a cocoa development project in the region around 2005, and some smallholders were still waiting to receive their seedlings 15 years later. Additionally, there was a history of vanilla growing, which stopped over a decade prior to this study due to challenges with crop purity. Previously, there were also farmer cooperatives, which gradually failed due to poor organisation.

##### Lasting Impacts from the Bougainville Conflict

The Bougainville Conflict took place for roughly a decade (1988–1998) and greatly impacted food systems during and after this period. During the conflict, cash cropping was completely halted, and most gardens were abandoned when locals moved to care centres to escape violence. Respondents stated that the food system has largely returned to its previous state since the end of the conflict, though there have been some lasting effects. For example, gardens are now usually planted closer to residences which may result in reduced fallow periods in areas closer to settlements. One respondent noted, “*We had gardens further away from the village [prior to the Conflict]. Now, we have fear. During the crisis, we had fear, so the distance to gardens is less now*”.

During the conflict, gardens were grown close to care centres, but locals were only able to visit gardens to harvest food roughly once a week and in the company of PNG security guards. During this period, which lasted four years, all respondents stated that they were short on food roughly two days in every seven.

The reduction in cocoa production has also outlasted the conflict, with respondents stating local production being nowhere near what it was prior to the conflict. As of 2002, Bougainville was producing roughly 20% of the cocoa it produced prior to the conflict [2]. Many respondents stated they had roughly half the amount of cocoa plants they had before the conflict, and production rates were higher per plant prior to the conflict as the cocoa pod borer had not yet infested the area.

While the environmental degradation of the mine at Panguna acted as a catalyst for the Bougainville Conflict, participants did not cite operations of the mine itself as a meaningful factor in gardening or cash cropping.

##### The Role of Gender in Food System Activities and Outcomes

Women play central roles in the food system, being primarily responsible for all subsistence activities and food preparation, as well as sharing responsibility for cash cropping activities and some animal-derived foods as detailed in Table 2. Respondents across all ages stated they had “gender equality” when asked if they felt equal to their partners in the home and regarding decision making. It is apparent that various community interventions promoting gender equality have occurred in the region (at the encouragement of several NGOs, religious groups, intergovernmental bodies, etc.). Younger respondents, particularly those under the age of 35, stated that they felt equal to their partners, and all age groups stated that this was improving due to educational and community interventions. Several local and external organisations have initiated female empowerment interventions in communities across Bougainville, including an overarching Bougainville Gender Investment Plan, which is a collaboration between the governments of PNG, Bougainville, and Australia [43].

Despite this emerging equality narrative, in practice gender roles and division of labour appear to be rigidly defined in the food system. For example, during community visits for this study, men were observed often sitting and telling stories while women were preparing food. If the men wanted coffee, they would ask the women to prepare the coffee so that they could continue to sit and tell stories. It was considered culturally inappropriate for the men to go where the women were working, or for the women to go where the men were sitting. The sentiment shared in a conversation with a male community member painted a picture of the wider perception of labour: “*I have a wife and many daughters, so there is not much for me do. Sometimes I clear the garden, but they always cook and go to the garden and take care of it*”.

#### 3.2.2. Environmental and Biophysical Components of the Food System

The main biophysical challenges cited in the food system are insects in the gardens, cocoa pod borer, floods, droughts, and continuous rain causing rot or fungal plant diseases. Most respondents indicated no major difference over time in soil quality, though some did suggest a reduction in the duration of fallow periods. All respondents cited no change in the availability of foraged foods, but did note an increased difficulty in hunting for bushmeat. The response was variable regarding river fish, though most indicated no significant change in availability of river fish over time. Most agreed that there was no change to marine fish supplies.

Cash cropping is another component of the food system that has changed substantially in the last half-century. Transport of crops has become increasingly difficult due to road damage. Respondents were previously able to sell cash crops and reported that now there are generally fewer methods of income generation due to transport restrictions. Though cocoa is still produced, wet beans are sold now instead of dry beans due to transportation challenges. This challenge is now compounded by damage induced by cocoa pod borer since about 2005. Consequently, the relatively long hours of labour required to harvest, ferment, and dry cocoa is no longer an efficient use of resources for farmers. As sale quantity is reduced and wet beans are a lower value item, income generation from cocoa is much less than what it was from the 1960s to 1980s. However, nearly all respondents still grow some cocoa, albeit in reduced quantities. An aid project distributed cocoa seedlings in the region in 2005, but many participants in the project stated that they were still waiting on roughly half of their seedlings. Many respondents also stated that betel nut production (a local, commonly used legal stimulant) had become a more reliable source of cash generation than cocoa.

In addition to the existing challenges that shape the food system, models predict that in the future, Bougainville and much of Melanesia will become warmer and wetter, extenuating problems that are already harming food security, namely an increase in crop damage from flooding and plant disease and pest or fungal damage [36,44,45]. Historically, blight has affected some crops in Bougainville, and wetter soils will worsen this problem. An increase in storm intensity may also damage crops.

### 3.3. Stresses and Shocks

**Figure 3:**
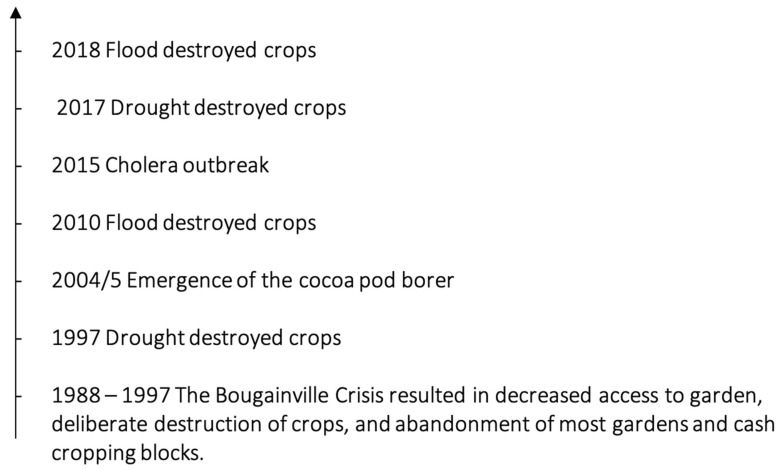
Timeline of stresses and shocks to the food system.

Global environmental change has contributed to several vulnerabilities throughout the Bougainvillean food system. These can manifest in numerous ways: from changes in the hydrological cycle caused by climate change resulting in crop damage, to decreased protein availability due to overhunting from high rates of population growth. Respondents noted numerous stresses and shocks as shown in the timeline in Figure 3. A detailed explanation of stresses and shocks are shown in Supplementary Table S2. The perception of vulnerability among respondents was mixed. Some respondents, particularly those in younger cohorts, were concerned about food procurement in the future due GEC drivers, though others shared the sentiment, “*We have plenty of land and good soils. We can always make more gardens. We can always make more food*”.

## 4. Discussion

In this study, a holistic food systems approach was taken to establish baseline food systems data in the case study area and use that baseline to identify potential vulnerabilities from stresses and shocks to these systems. We demonstrate that a more comprehensive systems approach that goes beyond the classical agricultural development paradigm raises issues potentially overlooked but essential for an improved, inclusive approach to “food systems development”. We outline the importance of social capital as well as the ability to identify and better understand interactions within these systems. Although there are robust existing resilience strategies within the studied food system, these may need more recognition to be strengthened to face future stresses and shocks.

Social capital, underpinning such activities as food sharing within clan systems, was prevalent in this study as a critical component within the case study food system. This aligns with Addinsall (2015), who demonstrated that socio-cultural assets are fundamental in the context of Melanesian food security [28]. Food sharing has persisted despite significant socio-political disturbances, particularly the Bougainville Conflict. Though this conflict disrupted the lives and livelihoods of nearly every Bougainvillean for a decade, the food system returned largely to its pre-conflict state in a remarkable manner, demonstrating the resilience capacity of the current system, which persists today.

The study area has remained a largely traditional system, though it is increasingly vulnerable to global environmental change as populations grow, land is degraded from overuse, and the climate gets warmer and wetter [36,37]. Interdisciplinary and holistic consideration of the food system is therefore necessary for food systems development and policy that achieves sustainability and resilience, including healthy human populations and ecosystems. Table 3 illustrates this necessity for a transition from classical agricultural development to the food systems development paradigm (FSDP). Consider, for instance, how the paradigm shift captures policy and project influence on gender roles in the area in designing food systems interventions. For example, as cash cropping tends to fall under the male domain, cash cropping projects without safeguards may alter gender power dynamics within households or fail to generate equitable incomes across the community. Another example shows that the classical agricultural development paradigm may suggest sub-optimal cash crops. For instance, vanilla may be a suitable cash crop in terms of its biophysical properties; however, inclusion of historical context indicates that vanilla has been associated with crop tampering, and so future vanilla market access may prove difficult due to reputational damage.

Despite the diverse set of resilience strategies in local food systems (detailed in Supplementary Table S3), participants in this study all cited food shortages in the last year.

An FSDP approach can shed light on food system interactions that may be difficult to measure or not be perceptible in the classical approach. For instance, an unquantifiable asset to the food system is the connection to place that the Bougainvilleans have, as is manifested in immeasurable ecosystem services and awareness of the environment. This connection is deep and a unique conceptualization of one’s surroundings. This was demonstrated, for instance, in the provision of cultivated and wild plants for cooking— not just for eating, but also for medicine and other practical uses, such as food wrapping (made from banana leaves), water storage (made from bamboo stalks), or skin and hair moisturizer (made from coconuts). With this acute awareness of place and knowledge of environmental factors, the community’s capacity to detect degradation and ecological vulnerability is heightened and can inform decision making, as was evident in the case of the Bougainville Conflict [29].

As these regions seek increased sovereignty, consideration of resilience and development strategies must consider this important factor, particularly in environments at risk for recurring conflict or that are otherwise sensitive to major socio-political shifts. The Autonomous Bougainville Government (ABG) is planning construction of a Level Three hospital in the study area, which will be accompanied by the construction of a road and have significant implications for connectivity. Taking an FSDP approach helps identify that this will likely result in a two-way increase in market interaction: locals will be able to access markets more easily, thus gaining more income from selling cash crops and market foods, but they will also have increased access to shop food. This may result in lower quality diets. Though current community member diets have only a small component of global connectivity, the contact and cultural assimilation of store-bought food has already occurred and may continue to become more prominent. This places the case study area in a unique position for longitudinal research, as it may provide the basis to understand how remote Melanesian communities and food systems respond to increased global connectivity, particularly as a function of increased sovereignty.

Early examples of success from the system-wide approach are beginning to emerge, such as the Food and Agriculture Organisation’s Flexible Multi-Partner Mechanism projects that are implemented both globally and on a national scale in countries across Asia, Africa and the Americas. These employ an approach that looks across the holistic food system and drivers therein to design interventions and minimize unforeseen consequences [49]. Widespread adoption of this approach is vital to ensure both healthy populations and a sustainable environment.

Situated in the context of a slowly recovering post-conflict Bougainville, and with the recent independence referendum, the region is at a pivotal point in its development which mirrors other parts of Melanesia and the Pacific that are also potentially seeking independence. It is hence timely for a transition to implement a food systems development approach in the region.

## 5. Conclusions

As food production is a means of nutrient acquisition, as well as a component of broader socio-cultural and ecological systems, it is vital that when designing food policy, programs, or aid projects that address food systems inadequacies in Melanesia, they are underpinned by contextual understanding. This must integrate disaster and conflict susceptibility, the impacts of global environmental change and socio-cultural factors, as well as climate suitability.

As the Autonomous Bougainville Government (ABG) continues to refine the ABG Strategic Development Plan and future policy development including moving forward with independence, it is important to reflect on historical susceptibility, notably as experience during the Bougainville Conflict, and consider socio-cultural elements of food systems. The historical susceptibility in the Bougainvillean context relates to inequity and land and water degradation. As these factors catalysed the rebellion that became the Bougainville Conflict, particular care should be taken in future development to ensure this historical susceptibility is not tapped again.

### Future Research and Limitations

We suggest that research needs to seek to better understand stakeholder needs and community context to inform food systems development projects. Numerous current projects emphasize the more classical agricultural development approach by reporting on progress with targets limited to infrastructure expansion, number of new plants (such as cocoa or coffee), women engaged, and income generation for participants.

The outcome of the referendum requires a gamut of policy building, which will necessitate improved information across all sectors. In the food systems literature, for example, further investigation into appropriate cash crops is needed and complete evaluation of suitable crops is outside of the scope of this research. Future studies need to be more holistic in their approaches and also cross disciplines, avoiding the siloes of agricultural research or public health research.

In a broader context, a framework for a systematic approach to crop selection orientated to equitable livelihoods rather than production should be explored. The discussion and results of this study illustrated why the FSDP approach is necessary to develop better policies and to improve food systems development projects. While a strategic approach to cash crop selection and decision making in the tropics would ideally have been applied to this study, no such framework currently exists. Establishment of such a framework specifically for the selection of cash crops that considers stakeholders and livelihoods, as well as environmental sustainability and cultural adoptability, should be pursued to better evaluate future opportunities for income generation through cash cropping.

The last identified area for future study in the arena of Bougainvillean food systems is into the complexities at play within gender dynamics. While superficially addressed in this study, there are numerous intricacies that field observations were unable to address in the current scope of this research. It is apparent that women and men alike are acutely aware of gender equality concepts, but despite numerous programs to engage women and the theoretical knowledge of equality in the community, it is far from realised in daily life.

## Figures and Tables

**Table 1:** Examples of shocks and stresses present in Melanesian food systems [2,4,14,15].

Theme	Shock	Stress
	**Recurring or Maintained Shocks can Become Stresses**	
Bio-physical	■ Natural disasters (floods, storms, landslides)	■ Increasing instance of pests and disease ■ Soil degradation, soil salination ■ Agri-biodiversity decline
Socio-economic	■ Political upheaval ■ Market forces ■ Interruption to market access (ex. due to COVID-19 or transport blocks such as road washouts)	■ Growing political tensions ■ Population growth ■ Market forces ■ Sustained barrier to market access (ex. due to COVID-19 or sustained road damage or high cost of fuel)

**Table 2:** Gender roles in the food system.

Labour Type	Men’s Role	Women’s Role
**Subsistence Farming**	Land clearing of land for new gardens, cutting trees, some weekly maintenance.	Weekly maintenance, harvesting, planting, weeding, digging, etc.
**Subsistence Decision Making**	Men are rarely involved in subsistence decision making.	Determining and actioning what is planted and harvested, garden size, etc.
**Cash Cropping**	Primary management and maintenance of all cash crops.	Management when no patriarch is present or occasional shared management.
**Cash Cropping Decision Making**	Some respondents stated that both parties co-managed cash cropping decision making and responsibility.	Some respondents stated that both parties co-managed cash-cropping decision making and responsibility.
**Fishing**	Both, though men tended to do more night fishing in the river as well as sea fishing.	Both, though women tended to do more daytime fishing in the river.
**Hunting**	Men only (on a roughly monthly basis).	Women did not participate in hunting.
**Food Preparation**	Killing of large animals for consumption and occasionally killing of small animals.	Food preparation was largely a female duty, though killing of small animals (namely chickens) was a shared responsibility. Due to the limited resources in these remote communities, cooking is very time-consuming.
**Foraging**	Men rarely forage.	Women forage often, frequently while en route to and from gardens.

**Table 3:** Challenges to the food system, how the classical agricultural development paradigms would address those challenges, and the illustration of how the food systems development paradigm addresses the challenge in a more thorough manner, more suited for the design of development projects.

Challenge	Agricultural Development Paradigm	Food Systems Development Paradigm (FSDP) Additional Consideration
**Environmental Factors**		
**Changing crop suitability and increased crop damage due to climate change**	Crop suitability given current biophysical factors.	Climate environment analogues and projected climate change modelling for crop suitability are needed for the FSDP approach: Ex. Crops should be assessed for appropriateness with inclusion of climate modelling outcomes.
**Increased vectors of diseases and pests (due to climate change and increased mobility**	Crop breeding and technical interventions.	The FSDP approach shows that beyond biophysical changes, climate change will also result in broader impacts to the food system, further amplifying current pest/disease challenges. Ex. Cocoa pod borer has impacted cash cropping in Bougainville, which may get worse with climate change.
**Socioeconomic and Cultural Factors**		
**Crop suitability**	Crop suitability given current biophysical factors.	Contextual factors for crops must be considered in the FSDP approach: Ex. In the Bougainvillean context, due to historical crop adulteration, Bougainville cannot access the vanilla market, so despite an appropriate climate, vanilla is an inappropriate crop to promote. Climate analogue should be considered in the FSDP approach: Ex. Crop selection should include modelling for future climate conditions.
**Increased pressure on natural resources and population growth**	Prioritisation of increasing efficiency, often through increased agricultural inputs.	Contextual factors for social systems must be considered in the FSDP approach: Ex. Changes in land tenure, changing social dynamics, and population growth are resulting in increased pressure on natural resources including soil, bush meat, and fisheries. This may result in changes to food sharing dynamics.
**Conflict**	Little consideration.	Social and historical factors for must be considered in the FSDP approach: Ex. Consideration of recent conflict will help to determine drivers of land pressure close to settlements and consider the vulnerability of social dynamics.
**Establish “Good Governance”**	Little consideration.	Political and historical factors for must be considered in the FSDP approach: Ex. Establishing “good governance” and “fiscal self-reliance” is required per the Bougainville Peace Agreement [34]. This means that conflict must be avoided, and sustainable and stable methods of food production are needed [46].
**Gender**	Prioritisation of increasing income for women.	Social gender-related factors for must be considered in the FSDP approach: Ex. Cash crops are largely in the realm of “male work” in Bougainville, so interventions in cash cropping may result in changes to gender dynamics.
**Human health**	Prioritisation of calories.	Health factors must be considered in the FSDP approach: Ex. Gardening, water-crossings on foot, and walking as the only method of transport as well as low access to food and processed or high calorie foods is correlated with low instances of disease (though it is noted that low access to medical care likely also limits access to diagnoses). An increase in income from cash crops may result in increased consumption of “shop food” (processed foods), which has sometimes led to an increased instance of metabolic disease in Melanesia [47,48].
**Taim hangre (hunger season)**	Prioritisation of calories.	Practical factors for must be considered in the FSDP approach: Ex. Food preservation in Bougainville is rare, but that there is public interest in methods for food preservation.

## Data Availability

Not applicable.

## Supplementary Materials

The following supporting information can be downloaded at: https://www.mdpi.com/article/10.3390/ijerph19084651/s1, Table S1. Description of the food system in Bougainville. Table S2. Explanation of shocks and stresses to the food system in Bougainville. Table S3. Resilience strategies that the community practices in response to shocks and stresses in the system (see Supplementary Table S2) in order to help achieve an adequate food supply. Figure S1. Images of Rich Picturing exercise. Each group drew their food system including food production, acquisition, and shocks and stresses on the food system. References [50,51,52,53] are cited in the supplementary materials.

## Appendix A. Selected Guiding Questions for Interviews

The go-along approach was used for interviews, however, some scripted questions were used to drive the conversation, some of which are highlighted below.

Small groups:

1. How many meals to you eat in an average day/week?
2. What do you typically eat for breakfast/first meal? For lunch/second meal? For dinner/third meal?
3. Where does your food come from?
4. Do you eat shop food? How often?
5. Who does the gardening?
6. Do you grow cash crops?
7. Who is in charge of cash crops?
8. What do you do with the money from cash cropping?
9. Who decides what you do with the money from cash cropping?
10. What has influenced what and how much you eat over your lifetime?
11. Have there been any events that have impacted what or how much you eat?
12. How often do you have an adequate food supply?
13. What do you do when you don’t have an adequate food supply?
14. Have you noticed any changes in what you eat over your lifetime?
15. Have you noticed any changes in how you procure or grow food over your lifetime?
16. Do you have any concerns about where you’ll get food in the future?

For garden tours:

1. Please talk me through what you grow in your garden (now and throughout the year) and how you grow it.
2. Does the composition of your garden change over time?
3. How long has this garden been here?
4. How long do you expect to have your garden here?
5. Why is your garden in this particular spot?
6. Where will your next garden be?
7. How do you cultivate the plants here?
8. How many different kinds of plants do you grow here?
9. Do you have any form of pest control?
10. Do you add anything to your garden to help it grow?
11. Do you irrigate this garden?
12. Over your lifetime, have there been any changes to the way you garden? If yes, please describe these changes.
