# Supplementary material for: Food Systems Development: The Necessary Paradigm Shift for a Healthy and Sustainable Agrarian Transition, a Case Study from Bougainville, Papua New Guinea
Source: Int J Environ Res Public Health. 2022 Apr 12;19(8):4651. doi: 10.3390/ijerph19084651 (PMC9029559; doi:10.3390/ijerph19084651)
Supplement: Supplementary file 1 [file ijerph-19-04651-s001.zip › ijerph-1581634-supplementary.pdf]

Supplementary Materials:

Supplementary Table S1. Description of the food system in Bougainville.

Food System Activity	Description	Stress/Shock	Social and Cultural Welfare and Livelihoods Element
**Subsistence crop production**	Rainfed, swidden agriculture with three to ten-year fallows. Several varieties of sweet potato, cassava, banana, taro (more abundant in higher elevation), yam, and singapore. Aibika, snake beans, cabbage, pawpaw, coconut, pit-pit, pumpkin tips, choco greens, corn, leafy green vegetables, breadfruit, citrus, and rambutans.	Continuous rain, flooding, drought, reduced land availability, pests, and crop disease.	Subsistence crop production from food gardens is primarily managed by the women, there is a very strong social system with food sharing when subsistence cropping is inadequate.
**Wild food gathering**	Wild greens (primarily fern, *posu'e*), cassava, yams, swamp taro, mushrooms, wild nuts (galip, tulep, borasidi), mangoes. Wild plants for medicinal usage, food storage or household utencils (ex. banana leaf food wraps, banana midrib cooking utensils, bamboo water containers).	Current harvest rates have had little impact on supply of wild food abundance, though as population pressure intensifies, overharvest may occur.	Ecosystem services from forests are vital to social and cultural practices. For example, bush medicine and kitchen accessories like food storage containers (ex. banana leaf). Gathering food and goods from the bush is conducted primarily by women.
**Hunting, trapping, and fishing**	River fishing for prawns, small fish (eaten whole) and large fish, some sea fishing, hunting of wild pigs, possums, giant rats, flying foxes, and large lizards.	Many of the older focus group participants suggested there has been a decline in the availability of bush meat since their childhood (1960s) due to over-hunting.	Fishing is conducted by men and women equally, though men tend to dive at night for fish or fish in the ocean while women do more pole and line fishing in the river during daylight hours. Hunting is conducted nearly solely by the men, though this is a relatively rare activity in the study area and more common in the mountainous region.
**Market**	Local surplus garden food and ocean fish sold at markets once each week.	Subject to availability of over-abundance of foods from subsistence food supply.	As families now need to pay school fees, health costs, and buy clothes and soap, the local and capital city markets are the primary local source of income outside of teaching, nursing, and police work. Due to the lack of refrigeration, market foods are only eaten one to two days a week.
**Store foods**	Rice, salt, coffee, tinned fish, sugar, and noodles, are transported by boat and foot from Buka, as well as sold out of canteens or through the "black market" (selling out of residences), which has limited availability and has a high cost beyond the means of most families.	Shop food is subject to cost/market volatility and is difficult to access due to lack of transportation and bad weather.	Store foods are a luxury to some and sharing of coffee, particularly on special occasions, is a popular social event.
**Cash Cropping**	Cocoa, copra, betel nut, and occasionally peanut.	Cocoa pod borer and transportation are the main challenges to the cash cropping system.	Men tend to manage cash cropping, though younger generations tend to experience increased female decision-making power.

Supplementary Table S2. Explanation of shocks and stresses to the food system in Bougainville.

Stresses and Shocks	Explanation
**Population Growth**	Many respondents stated that their gardens are larger now than in previous years due to larger family size. Fertility rates are high and thus population pressure an inevitable eventual stress on the food system. This will likely result in soil degradation, deforestation of primary forest for new gardens, and shortened fallow periods which will impair the ecological stability of the area and cause a positive feedback loop of ecological degradation.
**Extreme Weather Events due to Climate Change**	There are numerous and varied impacts of climate change on the Bougainvillean food system, the most often noted were continuous rain (consecutive days of rain during the wet season), floods, and more intense drought or longer dry seasons. These events repeatedly and frequently directly impact food access and food security by damaging parts of gardens and infrastructure, and in some cases destroying entire gardens.
**Human Disease Burden**	Tropical diseases are predicted to increase with climate change [44]. The most recent outbreak of cholera, for example, was in 2015 and impacted the local community's capacity for labour to maintain and procure food.
**Plant Disease and Pests**	Plant disease and pests may both increase with climate change, particularly in the tropics [45]. Pests are already frequently cited as a challenge and there are few existing strategies to reduce pest damage.
**Conflict**	Lasting impacts are felt from the Bougainville Conflict as noted.
**Distribution of Aid**	In 2004 – 2005 a major aid project distributed cocoa plants in the region in an effort to rehabilitate the area after the conflict. Participants stated that there was an uneven distribution of cocoa plants, however, and some locals are still waiting for their plants despite the fact that everyone paid the same fees. Though this does not appear to be a point of contention in the community at this time, it may become acrimonious as the region faces other increasing stresses.
**Urban Connectivity**	The study area has no transportation infrastructure and limited market access. As connectivity increases, a point of nutrition security arises. Traditional diets are rich in nutrients but low in sugar, and increase in connectivity will likely result in increased consumption of processed foods and sugar, which may act as an avenue to obesity, metabolic disease, and other problems associated with "cheap and easy" calories as has occurred in other parts of Bougainville [48].
**Loss in Agrobiodiversity**	Over the lifetimes of the oldest respondents, a reduction in agrobiodiversity was cited, particularly relating to staple crops. Participants noted an increased reliance on sweet potato in the last 20 – 30 years. The reduction in agrobiodiversity is two-fold: the amount of single staple food has intensified (transitioning to mostly sweet potato from a mixture of sweet potato, yam, cassava, and taro) as well as the number of varieties of a given staple, for example, while more than ten varieties of sweet potato were grown in the past, it is most common now for between five and ten varieties to be grown in a single garden.
**Overfishing**	Answers were mixed regarding the abundance of river fish. Usually, participants said there has not been significant change over time, indicating that the local population of river fish has not yet exceeded its maximum sustainable yield, though this may occur as human population increases.
**Overhunting**	It is widely known that mammals have been overhunted in Bougainville, though no respondents expressed worry due to this. It did not significantly impact the diets of those in the study area, who's primary animal protein sources were fish and chicken.
**Formalisation of Land Tenure**	Much of the customary land in Melanesia is transitioning to formalised land tenure [12]. As foraging predominantly occurs on customary land, this component of the food system may be challenged if land ownership policy is changed, particularly in as an effect of the upcoming referendum and potential change of governance.

Supplementary Table S3. Resilience strategies that the community practices in response to shocks and stresses in the system (see Supplementary Table S2) in order to help achieve an adequate food supply.

Existing Resilience Strategy	Explanation
**Food and Labour Sharing**	Social capital in Bougainville is significant, with a clan system and strong family ties. Clan members have gardens in various locations in the landscape: some are near the river while others are in elevated areas, so if one experiences a landslide, flood, or other disturbance, it is common to go and provide labour in another's garden and share food, or even to go to another's hamlet for meals. Clan members will also often bring food to each other's homes without a defined reason. Gifting of food is also common, particularly of fruit from determinate trees. For example, many rambutans ripened at once, so the excess was often shared around in communal environments or at social gatherings. There is a significant culture of religious worship, and food sharing at Sunday church services is a common event with many participants bringing food and sharing a meal after the service. The strategy of food (and sometimes labour) sharing was employed by all focus group respondents, who stated that food sharing was their primary source of nutrition when they experienced food shortages.
**Foraging**	Foraging is a significant component of the Bougainvillean diet. Going to the bush to collect ferns, wild nuts, mushrooms, and fruit is a common occurrence regardless of the availability of other foods. When gardens are destroyed or food is otherwise in short supply, respondents spent more time gathering and used this to supplement damaged crops. Participants stated that bush food is always abundant and as bush covers much of the landscape, it is resilient to localised damage. For example, if low elevation crops are damaged near the river, foraging can continue at higher elevations.
**Multiple Garden Sites**	Many locals have a primary food garden away from their hamlet, which is large and a smaller kitchen garden close to the residence. While the kitchen garden has reduced capacity to change locations in the same way as the primary food garden, having food production in multiple locations adds resilience to localised hazards like flooding.
**Increased use of Flood Tolerant Foods**	When an environmental hazard like a flood occurred, respondents noted an increase in foods that were less impacted, although located in or around food gardens (primarily bananas and coconut). As the edible portions of these plants are more resistant to flood damage, when gardens were destroyed, locals would temporarily increase intake of these foods as necessary.
**Staple Crop Diversity and Biodiversity**	The primary staple crop is sweet potato, of which respondents commonly grow between five and ten varieties. Additional staple crops are also quite diverse and include cassava (five to ten varieties), yam (three to five varieties), and banana (more than ten varieties), which allows for redundancy and thus resilience in the food system as well as more nutritious diets [40]. Sweet potato is a resilient crop that withstands a variety of abiotic and biotic stressors, requires low inputs, and is a particularly important crop for reducing food insecurity in the Pacific [50–52]. The biodiversity among these crops also facilitates resilience, namely pest resistance and sustainability of the farming system [53].
**Protein Diversity**	Protein sources are diverse and include chickens that are often raised near the home, river fish and prawns, ocean fish (usually purchased from the market), and bushmeat (usually wild pigs, giant rats, possums, flying foxes, or lizards). In the somewhat rare case that a wild pig is killed and found to have piglets, the piglets will then be raised and sold or consumed after maturity. These different sources are both wild and domestic; this variation in protein acquisition demonstrates redundancy in the system so that reliance or overexploitation of one element does not occur.
**Adoption of New Varieties**	Some respondents cited the adoption of new varieties, particularly of sweet potato or taro that were modified to ripen faster than old varieties or resist drought. These varieties were usually sourced from non-governmental organisations in Rabaul East New Britain, though this was rarely employed, likely due to the remote nature of the study area.
**Shop Food as "Back-Up"**	Albeit few, some respondents stated that they relied increasingly on shop food during periods of garden food shortage. Most respondents stated that they did not employ this strategy, though, as shop food is too expensive.

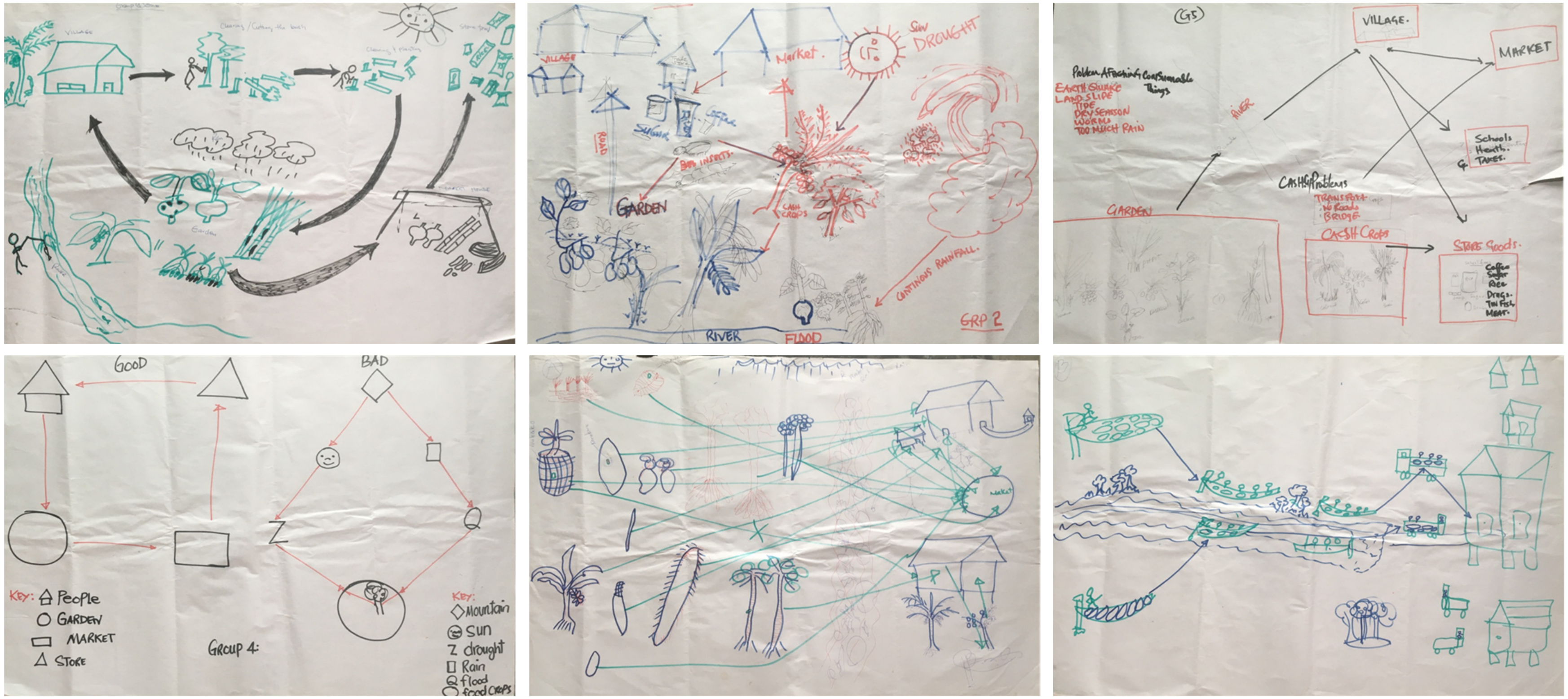

Supplementary Figure S1. Images of Rich Picturing exercise. Each group drew their food system including food production, acquisition, and shocks and stresses on the food system.
